# Peripheral Reproductive Organ Health and Melatonin: Ready for Prime Time

**DOI:** 10.3390/ijms14047231

**Published:** 2013-04-02

**Authors:** Russel J. Reiter, Sergio A. Rosales-Corral, Lucien C. Manchester, Dun-Xian Tan

**Affiliations:** Department of Cellular and Structural Biology, UT Health Science Center at San Antonio, San Antonio, TX 78229, USA; E-Mails: espiral17@gmail.com (S.A.R.-C.); lmanchester@stmarytx.edu (L.C.M.); tan@uthscsa.edu (D.-X.T.)

**Keywords:** oocyte, sperm, parturition, placenta, *in vitro*-fertilization + embryo transfer, ovarian follicle, melatonin, antioxidant

## Abstract

Melatonin has a wide variety of beneficial actions at the level of the gonads and their adnexa. Some actions are mediated via its classic membrane melatonin receptors while others seem to be receptor-independent. This review summarizes many of the published reports which confirm that melatonin, which is produced in the ovary, aids in advancing follicular maturation and preserving the integrity of the ovum prior to and at the time of ovulation. Likewise, when ova are collected for *in vitro* fertilization-embryo transfer, treating them with melatonin improves implantation and pregnancy rates. Melatonin synthesis as well as its receptors have also been identified in the placenta. In this organ, melatonin seems to be of particular importance for the maintenance of the optimal turnover of cells in the villous trophoblast via its ability to regulate apoptosis. For male gametes, melatonin has also proven useful in protecting them from oxidative damage and preserving their viability. Incubation of ejaculated animal sperm improves their motility and prolongs their viability. For human sperm as well, melatonin is also a valuable agent for protecting them from free radical damage. In general, the direct actions of melatonin on the gonads and adnexa of mammals indicate it is an important agent for maintaining optimal reproductive physiology.

## 1. Introduction

**Figure 1:**
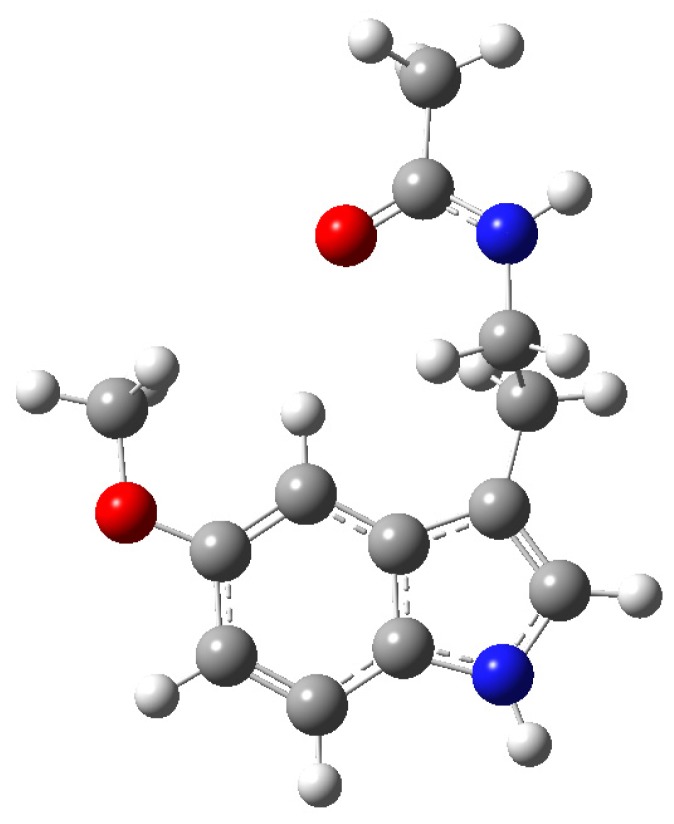
A three-dimensional view of melatonin (*N*-acetyl-5-methoxytryptamine), an indoleamine originally discovered to be a secretory product of the mammalian pineal gland and subsequently found to be produced in many different cells/organs and in all species of the plant and animal kingdoms. Melatonin easily crosses cell membranes and all morphophysiological barriers, e.g., the blood-brain barrier. While the current review summarizes its actions at the level of both the female and male reproductive system, the beneficial functions of this molecule probably extend to every cell in the organism.

Melatonin (Figure 1) is a heterogeneously-acting molecule with an exceptionally large skill set. Its unusual nature is emphasized by several of its recently uncovered features: (a) although discovered as a secretory product of the vertebrate pineal gland [1] and initially thought to be unique to that organ, it is now known to be produced in many, perhaps all, cells in the body [2–5]; (b) its first-described actions linked melatonin to circadian [6–9] and circannual [10–13] rhythm regulation, but subsequent studies validated the vast array of functions of this molecule, which include actions at the molecular level that change the physiology of organs and organisms [14–18]; (c) the indoleamine initially was thought to be produced only in animals that had a pineal gland [19], but it has subsequently been found to be synthesized in every living creature including bacteria [20,21] and unicells [22,23], and throughout the plant and animal kingdoms [24–26]; (d) melatonin works via well-defined membrane melatonin receptors [27–29], but its actions far transcend these receptors since binding sites have also been described in the nucleus [30–32] in the cytosol [33–35] and in the mitochondria [36] and, moreover, some of its actions, e.g., as a direct free radical scavenger [37–40], require no receptor (binding site) whatsoever, *i.e.*, these actions are receptor-independent [41–43]; (e) initially melatonin *per se* was thought to account for all the observed actions associated with this indoleamine; however, melatonin is also a pro-drug [44] in that its metabolites have actions like the parent molecule itself [45–48] and isomers of melatonin have been discovered (to date, exclusively in plants) [49–51] whose actions remain undefined.

Based on what is described in this brief introduction, it is probable that no cell or function in either the plant or animal kingdoms totally escapes the impact of melatonin. Research especially within the last two decades has certainly borne this out, data that is available in numerous published reviews [52–60]. Summarized herein is only a miniscule amount of the extensive published data set. Topics selected for inclusion were those not recently covered in depth in other surveys. Emphasis is on the beneficial effects of melatonin, which seem to be the only actions this indoleamine has, on those organs/situations selected for inclusion in this review.

## 2. Melatonin: Improving Peripheral Reproductive Health

Historically, when one thought about the role of melatonin in terms of its impact on reproduction it related to the ability of the seasonally-changing melatonin signal to control annual fluctuations in reproductive capability in what is referred to as photoperiodic species [12,61,62]. Thus, as day length changes on a seasonal basis, the duration of the daily nocturnal rise in melatonin is similarly influenced [63]. The nightly change in the duration of elevated melatonin provides seasonal breeders with time-of-year information and allows them to anticipate the upcoming season and to alter their reproductive physiology accordingly [64–66] to ensure delivery of the offspring at the time of year maximally conducive to their survival, *i.e.*, typically in the spring [13]. The regulatory processes governing the ebb and flow of the hormones from the hypothalamopituitary axis that control reproductive capability in these seasonally-breeding species involves actions of melatonin in the basal hypothalamus and the pars tuberalis of the anterior pituitary gland which, in turn, determine the release of hypophyseal gonadotropins [67,68]. Since humans are continuous as opposed to seasonal breeders, changing seasonal day lengths have no or a very minor influence on reproductive efficiency throughout the year. This does not mean, however, that melatonin is inconsequential in terms of the reproductive organs of humans. As noted here, because of data uncovered within the last two decades it has become increasingly apparent that melatonin has multiple effects directly at the level of the gonads and their adnexa in the human and other mammals. As summarized below, these actions seem to be highly beneficial to the maintenance of optimal function of the reproductive system.

## 3. Melatonin: Female Reproductive Health

### 3.1. Ovary

**Figure 2:**
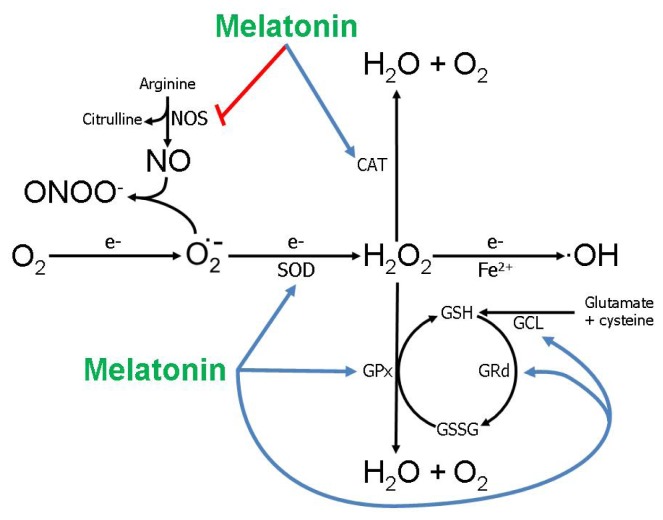
This figure illustrates the actions of melatonin in reducing free radical-mediated molecular damage. Melatonin stimulates (blue lines) several antioxidative enzymes including superoxide dismutase (SOD), glutathione peroxidase (GPx), glutathione reductase (GRd) and glutamylglycine ligase (GCL). It also inhibits (red line) the pro-oxidative enzyme nitric oxide synthase (NOS). In addition to modulating the activity of these enzymes, melatonin directly scavenges the highly toxic hydroxyl radical (·OH), the peroxynitrite anion (ONOO^−^) and possibly some other radical and non-radical products. The superoxide anion radical (O_2_^•−^), hydrogen peroxide (H_2_O_2_) and the ·OH are referred to as reactive oxygen species (ROS); nitric oxide (NO) and ONOO^−^ are referred to as reactive nitrogen species (RNS). O_2_ = molecular oxygen; e^−^ = electron; Fe^2+^ = ferrous iron.

As in other organs, some actions of melatonin on the ovary rely on conventional membrane receptors (MT1/MT2) and possibly on the nuclear receptor RZR/ROR superfamily, although for the latter the data are meager. The signal transduction cascade of the MT1/MT2 receptors often result in the inhibition of cAMP which leads to a downstream reduction in protein kinase A [29,69]. While this is a general rule, it is often violated since these receptors are commonly associated with other signaling pathways [70,71]. In relation to the ovary, the MT1 and MT2 receptors have been specifically localized on the granulosa and luteal cells in the human [72,73] and in the antral follicular cells and corpus luteum of the rat [74]. In addition to these receptor-mediated actions, congruent with other tissues, melatonin influences the physiology of all ovarian cellular components via stimulation of antioxidative enzymes and its multifaceted free radical scavenging activities [58], actions that are obviously receptor independent (Figure 2).

As in other body fluids, melatonin is also detectable in ovarian follicular fluid at concentrations, at least in the human, that exceed those in simultaneously obtained blood samples. According to Brzezinski and colleagues [75], the daytime radioimmunoassayable melatonin concentrations in the Graafian follicular fluid of women undergoing *in vitro* fertilization-embryo transfer (IVF-ET) are more than 3-fold higher than in the circulation (36.5 *vs*. 10.1 pg/mL, respectively). These observations were confirmed by Ronneberg *et al.*[76] who also observed that the values of melatonin in follicular fluid collected from women going through IVF-ET were also higher than in the peripheral blood with the ratio of follicular fluid to blood levels being essentially identical to those reported by Brzezenski *et al.*[75]. When levels were compared from samples collected during summer (referred to as the light period) and dark period (winter) (this study was conducted in Oulu, Finland, about 4° south of the Arctic Circle), day time levels of melatonin in the follicular fluid were slightly higher in the dark season samples. There was no correlation between melatonin levels and the volume of fluid (a rough estimate of follicular size) collected from individual follicles nor did the melatonin levels correlate with estradiol, progesterone, testosterone or prolactin levels in the serum.

A more detailed analysis of follicular fluid melatonin concentrations in humans showed that the levels were two times higher in large human follicles just prior to ovulation compared to those in smaller antral, immature follicles [77]. The authors of this report speculated that because melatonin and its metabolites are such potent antioxidants [40,46,47], the elevated melatonin concentrations in the follicular fluid at the time of ovulation would be physiologically advantageous. This is because the ovulatory process has been likened to inflammation, which is associated with high free radical production [78].

The fluid aspirated during the day from 3 to 8 mm diameter porcine ovarian follicles also contains radioimmunoassayable melatonin at a concentration of roughly 10^−11^ M [79]. These values are somewhat lower than those generally measured in the human ovarian follicular fluid. The authors also showed that 10^−9^ M melatonin enhanced *in vitro* maturation of porcine oocytes as well as their parthenogenetic embryonic development.

**Figure 3:**
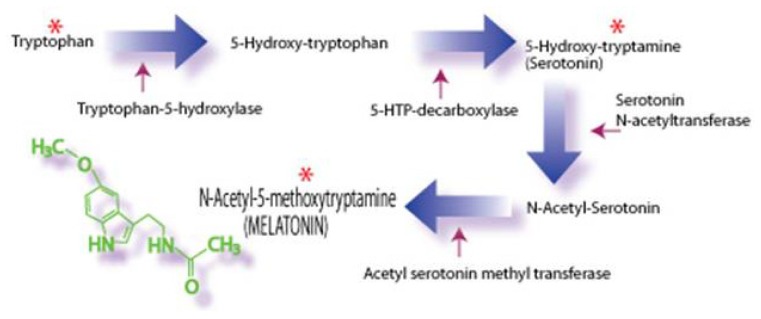
Schematic representation of the synthesis of melatonin from the amino acid, tryptophan. Tryptophan, which is taken up from the blood, via the four step pathway outline is converted to *N*-acetyl-5-methoxytryptamine (melatonin). Melatonin is best known for its production in the cells of the pineal gland from which it is quickly released in body fluids, *i.e.*, blood and cerebrospinal fluid. Circulating melatonin has both receptor-mediated and receptor-independent actions. Many other cells also produce melatonin; in this case, the indoleamine does not gain access to the blood in any appreciable amounts but rather works near its site of synthesis as an autacoid or as a paracoid.

The origin of melatonin in the follicular fluid was commonly thought to have been exclusively a result of its uptake, against a gradient, from the blood [80]. There are, however, other ovarian cells that synthesize melatonin which could obviously release it into the follicular fluid. Thus, the final enzyme in the melatonin synthesis pathway, *i.e.*, ASMT (Figure 3), has been identified in the cumulus cells of the bovine follicle suggesting that these cells are capable of melatonin production [81]. While there is still debate regarding this issue, ASMT is often believed to be the rate-limiting enzyme in melatonin synthesis [82,83]. If melatonin is produced in bovine cumulus cells, it is unlikely that this would be a feature of a single species; thus, it is easy to imagine that melatonin in the follicular fluid is derived from more than a single site.

**Figure 4:**
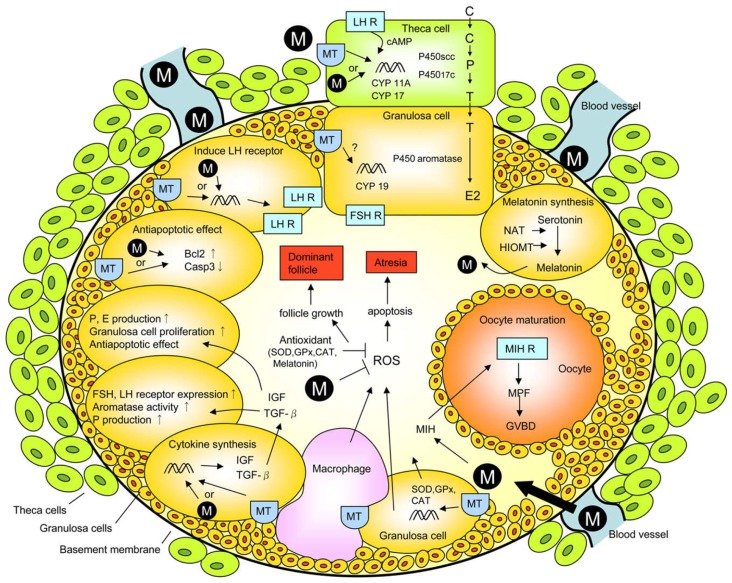
In the ovarian follicle, melatonin (represented in this figure by the M) impacts the function of numerous cells, especially granulosa cells and the ovum (oocyte). The actions of melatonin in these cells are mediated via membrane receptors (MT, in particular MT1 and MT2) and also possibly via binding sites in the nucleus and in the cytosol. In addition to its receptor-mediated actions, melatonin also functions as a direct free radical scavenger to reduce oxidative stress at the level of the ovary; this beneficial action is carried out without an interaction with a receptor. Additional antioxidant functions of melatonin are achieved when the indole stimulates enzymes which metabolize free radicals to less toxic products. The antioxidative enzymes include superoxide dismutase (SOD), glutathione peroxidase (GPx) and catalase (CAT) in thecal cells, granulosa cells and in the follicular fluid. Via these actions, melatonin reduces free radical damage, which would be especially bad for the ovum, and maintains these elements in an optimally functional state. The origin of melatonin in the follicular fluid is the blood and from its local synthesis in granulosa cells. C, cholesterol; LH R, LH receptor; FSH R, FSH receptor; NAT, *N*-acetyltransferase; HIOMT, hydroxyindole-*O*-methyltransferase (currently known as acetylserotonin methyltransferase, ASMT); MIH, maturation-inducing hormone; MPF, maturation-promoting factor; GVBD, germinal vesicle breakdown; ROS, reactive oxygen species; IGF, insulin-like growth factor; TGF-β, transforming growth factor β. From Tamura *et al.*[80].

The inflammatory-like processes identified in the ovary at the time of ovulation include augmented synthesis of prostaglandins and cytokines, increased activation of proteolytic enzymes and elevated capillary permeability, features that are associated with a high production of damaging reactive oxygen species (ROS) [84–86]. Moreover, macrophages, leucocytes and vascular endothelial cells, all of which are in the vicinity of large follicles [87] contribute free radicals at the time of follicle rupture [88]. Thus, to protect the ovum from oxidative damage during its expulsion from the ovary, the presence of melatonin would ensure that it escapes molecular mutilation thereby ensuring a healthy embryo and fetus. The multiple actions and interactions of melatonin at the level of the ovarian follicle are summarized in Figure 4.

While the methodologies for assisted reproductive technologies, e.g., IVF-ET, have improved steadily within the last two decades, poor oocyte quality remains a major problem. This is generally believed to be a result of oxidative damage to the gamete [89]. Radical scavengers as well as antioxidative enzymes, which metabolize ROS to innocuous products, are essential in protecting ova from oxidative damage. Since melatonin is both a direct free radical scavenger [37,90,91] and it also stimulates gene expression and activities of antioxidative enzymes [92–94] (Figure 2), Tamura *et al.*[95] reasoned that the indoleamine may have utility in improving the quality of human ova used for IVT-ET.

In the initial aspect of their study, follicular fluid was sampled at the time of transvaginal oocyte retrieval and examined for its levels of 8-OHdG (8-hydroxy-2-deoxyguanosine, a product resulting from free radical damage to DNA). The level of this damaged product in the intrafollicular fluid was correlated with the degenerative state of the ovum collected from the same follicle [95,96]. The amount of damage to the oocyte was judged on the basis of their morphology, in particular the darkened, vacuolated and irregular ooplasm. The levels of 8-OHdG were positively correlated with the degree of degeneration apparent in the ovum and, intriguingly, the levels of the damaged DNA product was negatively correlated with the intrafollicular concentrations of melatonin, a potent antioxidant. These two observations suggested to the authors of the report that the degenerative status of the ovum was a consequence of excessive free radical damage and potentially involved the diminished protection due to the depressed melatonin values.

**Figure 5:**
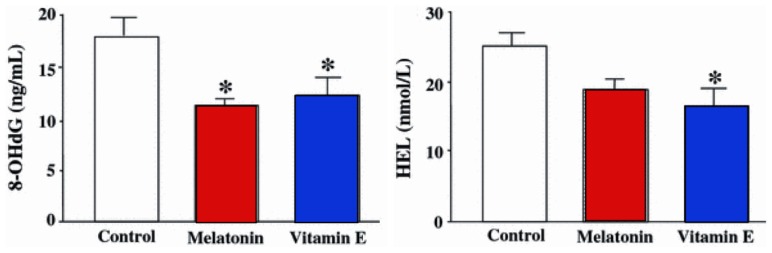
Mean (±SEM) 8-OHdG (8-hydroxy-2-deoxyguanosine; a product of oxidatively damaged DNA) and HEL (hexanoyl-lysine adduct; a product of oxidatively damaged lipid) levels in the follicular fluid of women treated with melatonin (3 mg daily) or vitamin E (600 mg daily) for 37 days. Both antioxidants obviously reduced free radical damage; however, even though the dose of melatonin was 200 times less than that of vitamin E, it proved as effective in reducing free radical damage to the oocytes. * *p* < 0.05 compared to control values. Modified from Tamura *et al.*[96].

To test this possibility, 18 patients undergoing IVF-ET who had failed to become pregnant during a previous IVF-ET cycle were given either melatonin (3 mg daily) or vitamin E (600 mg daily) from the fifth day of the previous menstrual cycle to the time of oocyte/follicular fluid retrieval [96]. The levels of 8-OHdG and HEL (hexanoyl-lysine adduct, a biomarker of lipid peroxidation) were then measured in the follicular fluid and compared with the concentrations of these constituents measured in the earlier cycle. Both the damaged DNA and lipid product was found to be lower as a result of treatment with the antioxidants (Figure 5). The obvious implication of these observations is that antioxidants could be used to reduce molecular damage in ovarian follicles, likely including the ovum, and that their use would improve the success of pregnancy during IVF-ET procedures.

Finally, to test the clinical utility of melatonin in enhancing fertilization and pregnancy success during IVF-ET, Tamura and co-workers [96] selected 115 patients who had failed to become pregnant in a previous IVF-ET trial and who had a fertilization rate below 50%. Fifty six of these women were given melatonin (3 mg/day) and 59 served as untreated controls. Melatonin treatment markedly improved the fertilization rate and 11 of 56 patients achieved pregnancy. By comparison, for the 59 women who did not receive supplemental melatonin, the fertilization rate was unchanged (compared to that in the previous IVF-ET cycle) and only 6 of 59 patients became pregnant (Table 1).

**Figure 6:**
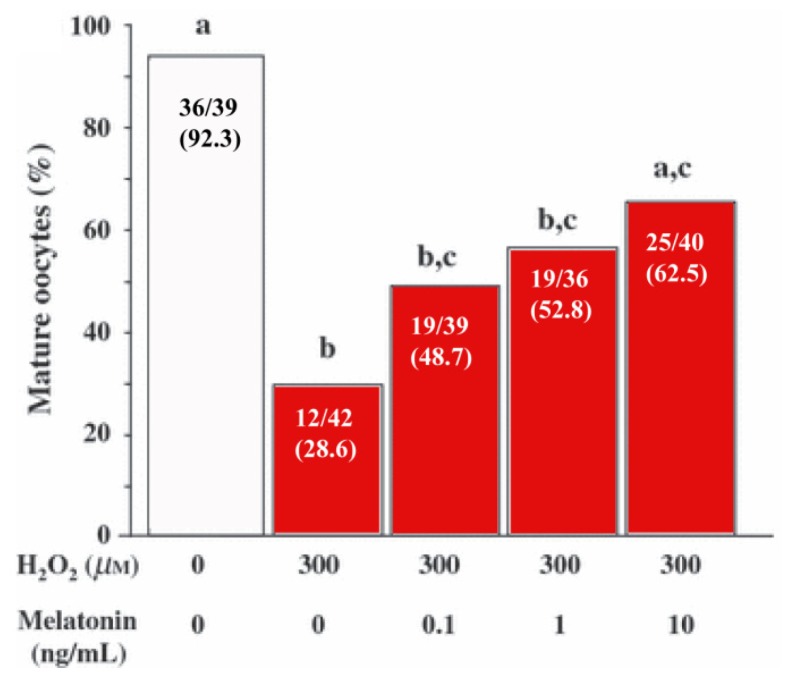
The *in vitro* exposure of denuded mouse oocytes to the oxidizing agent, H_2_O_2_ (300 μM), caused a marked drop (from 92.3% to 28.6% in the percentage of cells that matured (presence of first polar body; MII stage oocytes) within 12 h. Increasing concentrations of melatonin in the medium clearly improved the percent of H_2_O_2_-exposed oocytes that reached maturity. The number of oocytes in each treatment group varied from 36 to 42. Different letters indicate statistically significant differences between groups (*p* < 0.01; Chi-square test). Modified from Tamura *et al.*[96].

Tamura *et al.*[96] further examined the actions of melatonin on oxidative stress and quality of oocytes collected from mice induced to ovulate using pregnant mare’s serum. Oocytes, denuded of their cumulus cells, were incubated *in vitro* with the oxidizing agent, H_2_O_2_, without or with increasing concentrations of melatonin (0, 0.1, 1.0 or 10.0 ng/mL). The number of oocytes that reached maturity (as indicated by the presence of the first polar body; metaphase II [MII] stage oocytes) were then counted. Increasing concentrations of melatonin reduced the toxicity of H_2_O_2_ (free radical damage) and clearly elevated the percent of oocytes that reached maturity (Figure 6).

Collectively, the data of Tamura and colleagues [95,96] documents that oral melatonin supplementation increases the concentrations of this antioxidant in human ovarian follicular fluid. Moreover, this leads to improved fertilization and pregnancy rates in women who had previously had difficulty in achieving pregnancy during IVF-ET. The protective actions of melatonin are likely related to the ability of this molecule to scavenge free radicals and elevate antioxidative defense mechanisms in the oocyte.

**Figure 7:**
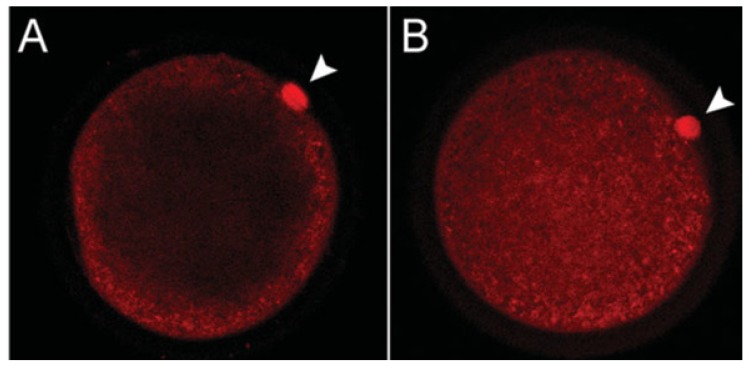
Distribution of mitochondria (identified with Mito Tracker^®^ Red) in MII bovine oocytes matured in the absence (**A**) or presence (**B**) of melatonin in the culture medium. The medium was supplemental with melatonin at a concentration of 10 ng/mL. The subcellular distribution of the mitochondria was markedly impacted by the presence of melatonin, probably due to the actions of the indoleamine or the cytoskeleton. In the control cells, the mitochondria were clustered around the periphery of the ovum whereas melatonin treatment caused their dispersion throughout the cell. The arrowheads identify the first polar body. From El-Raey *et al.*[97] with permission.

In cultured bovine oocytes, melatonin was also shown to promote maturation [97]. When cumulus-oocyte complexes from abattoir-collected bovine ovaries were incubated with melatonin, the indoleamine, especially at concentrations of 10–50 ng/mL, enhanced the rate of oocyte maturation (to MII stage). The same concentrations of melatonin also augmented the percentage of oocytes that had fully expanded cumulus cells, an important marker of oocyte maturation [98] and a requirement for fertilization and blastocyst development [99]. These alterations occurred in the absence of significant changes in either progesterone or estradiol production by the cumulus cells. While melatonin seemed not to substantially alter mitochondrial activity, it had a marked effect on the distribution of mitochondria (identified with Mito Tracker^®^ Red) in bovine *in vitro* matured oocytes. Thus, in control non-melatonin treated oocytes, the mitochondria were distributed almost exclusively in what the authors described as the subcortical region; in contrast, after melatonin treatment the mitochondria were found throughout the MII oocyte (Figure 7). The specific significance of the two widely different patterns of mitochondria distribution in oocytes was not revealed in this study. Since microtubules are known to be involved in the movement of mitochondria within cells El-Raey and co-workers [97] surmised that melatonin had cytoskeletal actions in bovine oocytes, an assumption that is certainly consistent with other studies where melatonin has been shown to modulate cytoskeletal organization [100].

Because of melatonin’s obvious antioxidative capabilities, El-Raey [97] also estimated ROS formation, as indicated by H_2_O_2_ fluorescence, in culture bovine oocytes. Judging from the fluorescence intensities, oocytes not cultured in medium containing melatonin had significantly higher levels of H_2_O_2_ compared to oocytes exposed to melatonin. This is an important finding since it argues strongly that oxidative damage, which would compromise their maturation, was also reduced in the oocytes. Whether the drop in H_2_O_2_ fluorescence as a consequence of melatonin exposure was due to the direct scavenging actions of the indoleamine, e.g., on the superoxide anion radical (Figure 2), or because it reduced electron leakage from the respiratory chain and thereby lowered ROS formation [101] was not determined.

Besides protecting follicular cells and the ovum from oxidative stress, melatonin may actually hasten the growth and maturation of ovarian follicles. When cultured caprine ovarian fragments containing primordial follicles were incubated in medium containing both melatonin (concentrations ranging from 100 pM to 1 nM) and follicle stimulating hormone (FSH; 50 ng/mL), folliculogenesis proceeded at a more rapid rate when compared to tissues treated with either agent alone [102]. After the addition of both melatonin (1 nM) and FSH, follicle size and ovum diameters were increased above those of other treatment groups after 7 days of incubation. The integrity and viability of the follicles were confirmed using ultrastructural techniques and appropriate fluorescent probes.

Continual-release subcutaneous melatonin implants have also been found to advance ovarian follicular development *in vivo* in dromedary camels [103]. In this study, follicular size was monitored using ultrasonography. The camels were treated with melatonin or placebo implants two months prior to the normal breeding season and ultrasound evaluations were made at weekly intervals thereafter. Throughout the evaluation period, follicular enlargement proceeded at a faster rate in the melatonin-treated animals compared to the controls; at 7 weeks, 5 of 6 camels given melatonin had ovulatory-sized follicles (≥10 mm) while none of the controls had follicles of that size. All the melatonin-implanted camels ovulated and after exposure to virile stud males, two became pregnant. The authors concluded that a continual-release subcutaneous melatonin implant is a useful tool to advance the breeding season of the dromedary camel. The findings in the camel are in accord with a substantial body of published literature which documents the positive effects of melatonin in advancing the breeding season and improving fecundity in large domestic mammals [104–107].

Ideas related to whether melatonin has direct or synergetic actions with other hormones on steroidogenesis in granulosa or luteal cells are in a state of flux. In the recent report of El-Raey *et al.*[97], melatonin supplementation during *in vitro* maturation of cumulus-oocyte complexes was without effect on the amount of progesterone produced. This is in accordance with some other reports where melatonin did not influence progesterone production [108]. On the other hand, melatonin reportedly inhibited [109,110] or elevated [111] progesterone under similar circumstances in yet other situations. Relative to the action of melatonin an ovarian estrogen production, the data are equally as inconsistent [97,112–114]. The highly variable responses to melatonin in these *in vitro* situations may be attributable to any of a variety of factors such as the cell type examined, the experimental model used, the species of origin of the studied cells, and the dose or duration of melatonin treatment [80,115,116]. It is virtually impossible to perform such studies *in vivo* because of the origin of the steroids from several different sources.

Ovary transplantation is still in the experimental stage since, possibly due in part to the complex nature of this organ, the tissue generally does not grow well after cryopreservation and thawing. Any organ that is transplanted suffers from ischemia/reperfusion injury as a result of excessive ROS generation and the associated oxidative stress. Given the ability of melatonin to directly detoxify free radicals, Hemadi and collaborators [117] tested the ability of melatonin to improve the maturation of transplanted murine ovaries. Vitrified ovaries obtained from 10-day-old mice and incubated with melatonin (100 μM) were thawed and subcutaneously transplanted into adult ovariectomized mice of the same strain [118]. After grafting, the recipient mice received a daily injection of melatonin (20 mg/kg) for 2 days. Ovarian grafts were harvested at several intervals over the following 32-day period. Clearly, incubating ovaries in a melatonin-enriched solution prior to their implantation followed by a melatonin injection into the recipients for 2 days thereafter improved the histological and the immunohistochemical parameters of the transplanted tissue. The authors judged the quality of the cumulus-oocyte complexes to be improved in the developing follicles and there was a marked increase in the number of follicles that underwent maturation. Revascularization of the transplants seems not to have changed. Had the *in vivo* melatonin injections been continued beyond 2 days, however, it is possible that ovarian grafts would have been further improved and better revascularization may have occurred. The outcome of this study is consistent with reports with other organs where morphological and functional improvement was obvious when melatonin was added to the preservation fluid prior to organ transplantation.

### 3.2. Placenta

The placenta is a critical component of the life support system of the fetus. For the fetus to develop normally, the placenta must function optimally. Considering melatonin’s protective actions due to its antioxidant and anti-inflammatory functions, it would be advantageous for the fetal/placental unit to produce melatonin for its local use.

As already pointed out, melatonin is no longer considered to be derived uniquely from the pineal gland. Surgical removal of the pineal, although obliterating the circadian blood melatonin rhythm, is not accompanied by depletion of the indole from other organs. In some cases, melatonin concentrations actually increase in subcellular organelles of other cells after pineal gland removal [119]. Also, while the circadian production and release of melatonin from the pineal gland is well documented [120–122], other organs including the retina [123] and harderian gland [3] continue to synthesize melatonin in a circadian manner in the absence of the pineal gland. What is characteristic of melatonin in organs other than the pineal gland is that it is not released into the general systemic circulation, at least not in any appreciable amounts [124,125]. Rather, melatonin in extra-pineal organs presumably is used locally as an autocoid or as a paracoid [126]. As with melatonin synthesis, the receptors for this hormone are much wider spread than originally envisaged [27,29,127]. After a recent comprehensive survey of the literature on the distribution of peripheral (non-neural) melatonin receptors, it was obvious that few tissues lack them [127]. Moreover, the reason membrane melatonin receptors appear to be absent from some tissues may be merely because no one has looked for them at these sites. Even if there are some cells that are actually devoid of the classic membrane melatonin receptors (MT1 and MT2) as well as other binding sites for the indole would not mean that melatonin has no actions at these locales, since melatonin’s receptor-independent functions in free radical detoxification would still exist [128,129].

In 2005, Iwasaki *et al.*[130] reported the presence of mRNA transcripts for alkylamine *N*-acetyltransferase (AANAT), sometimes considered the rate-limiting enzyme for melatonin synthesis [131], and acetylserotonin methyltransferase (ASMT), the melatonin-forming enzyme [132], in placental tissue obtained during the first trimester of human pregnancy. DNA sequencing of the RT-PCR products were found to be identical for the genes for AANAT and ASMT. The authors surmised that melatonin produced locally works in a paracrine manner to enhance the function of placental tissue. One action that they showed melatonin to mediate at the level of the trophoblast cells was the augmented release of human chorionic gonadotropin (hCG). Since they also documented the presence of RNA transcripts for the MT1 and MT2 melatonin membrane receptors in placental tissue, the action of melatonin on hCG release was assumed to be mediated by these receptors although no direct proof for this was provided.

A much more comprehensive study extended the previous findings related to melatonin synthesis by the human placenta; in this case the placental tissue was collected at full term after vaginally-delivered fetuses [133]. With a combination of methods that included RT-PCR, western blots and a radiometric assay, AANAT and ASMT were localized in villous trophoblasts. The authors also characterized the classic membrane receptors (MT1 and MT2) as well as the retinoid-related orphan nuclear receptor alpha (RORα) melatonin receptor in both mononucleated cytotrophoblasts and in the multinucleated syncytiotrophoblast syncytium. Autocrine and/or paracrine actions of melatonin on these cells were proposed.

A balance between the formation of the syncytiotrophoblast syncytium from cytotrophoblasts and its degeneration via apoptosis is necessary to prevent pathologies from developing in the placenta. Melatonin, which as pointed about above, is produced in the cytotrophoblasts [130,133], has a prominent regulatory effect on apoptosis. It has been repeatedly demonstrated that melatonin exhibits anti-apoptotic actions in normal cells while being pro-apoptotic in cancerous cells [134]. These dual functions are believed to be exploited by the placenta to maintain a balance between the villous cytotrophoblasts and the syncytiotrophoblast [135].

**Figure 8:**
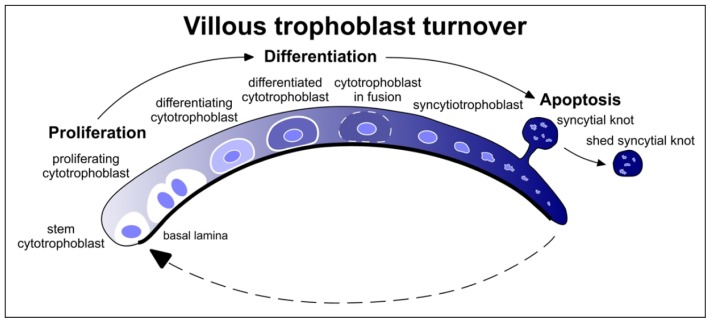
Turnover of cells that make up the villous trophoblast is essential for proper functioning of the placenta. Proliferative cytotrophoblast stem cells differentiate and eventually exit the cell cycle. These cells then fuse to form a multinucleated syncytium, the syncytiotrophoblast. Within several days cells of the syncytiotrophoblast undergo apoptosis. The additions of differentiated cytotrophoblasts replace the syncytiotrophoblast cells that are lost via apoptosis. The apoptotic loss of the syncytiotrophoblast is balanced by the fusion of differentiated cytotrophoblast cells. Locally produced melatonin seems to be involved in maintenance of homeostasis by limiting apoptosis of the differentiated cytotrophoblasts while enhancing apoptosis of the syncytiotrophoblast; these latter cells have characteristics of cancer cells in which melatonin also causes apoptosis. From Lanoix *et al.*[135].

As diagrammatically represented in Figure 8, the proliferating villous cytotrophoblasts (stem cells) differentiate and fuse into the syncytiotrophoblast, which is non-proliferative and undergoes rapid apoptosis [136]. Thus, during a normal pregnancy the syncytiotrophoblast turns over and is continually renewed. A precise balance between the fusion of the villous cytotrophoblasts into the syncytiotrophoblast is required to prevent placental pathology. Given the actions of melatonin in regulating apoptosis in normal cells (in this case, the cytotrophoblasts) and causing apoptosis in cancer-type cells (in this case the tumor-like syncytiotrophoblast), the indole could have a major influence in creating a stable villous cytotrophoblasts/syncytiotrophoblast homeostasis.

Additional evidence also suggests that locally-produced melatonin may be important for maintaining a healthy placenta. Throughout pregnancy, mononucleated stem cells, the cytotrophoblasts, rapidly proliferate and differentiate into either villous or extravillous cytotrophoblasts. The latter invade the uterine wall and remodel this tissue and the spiral arteries. The villous cytotrophoblasts, on the other hand, continue to proliferate, differentiate and fuse to form a syncytium, the multinucleated syncytiotrophoblast [136]. This syncytial tissue is non-proliferative and rapidly undergoes apoptosis [137]. The development and transformation of villous cytotrophoblasts make them reminiscent of tumor cells [138] and they have actually been referred to as having pseudo-tumorigenic properties [139].

Experimental findings support the proposed role for melatonin in maintaining a balance between syncytiotrophoblast formation and degeneration. In culture, melatonin was found to inhibit villous trophoblast apoptosis [135]. This action involved the MT1 and MT2 membrane receptors for the indoleamine. Western blot analysis established that melatonin reduced both the Bax/Bcl-2 intrinsic apoptotic pathway and caspase-9 expression in the villous trophoblast cells. Similar studies were performed using choriocarcinoma cells (BeWo), which form a syncytium [140] and are often used as a model of the syncytiotrophoblast. The BeWo cells also produce melatonin and express its membrane receptors as well as the RORα receptor [133]. In this model, melatonin promoted apoptosis, again via a membrane receptor-mediated pathway. These findings are as predicted based on studies using other normal and cancerous cells [134]. Importantly, since BeWo cells are a good model of the syncytiotrophoblast, the findings highlight the likely involvement of melatonin, which is produced in the cells in question, in ensuring a stable cytotrophoblasts/syncytiotrophoblast relationship throughout pregnancy.

A faulty or abnormally functioning placenta is known to contribute to a number of disease states that impact both the fetus and the mother. While the discussion of these conditions is generally considered to be beyond the scope of this review, the reader may want to consult other sources of information [95,141–143], especially considering that melatonin has been shown to be beneficial or is predicted to be beneficial in the treatment of these conditions. Of special interest is pre-eclampsia, intrauterine growth restriction, fetal hypoxia/anoxia, leukoplakia, and abortion.

### 3.3. Amnion and Amniotic Fluid

There are several reports related to melatonin in the amniotic fluid. The first publication on this subject claimed unexpectedly high concentrations of melatonin in fluid collected via amniocentesis at weeks 35–39 of pregnancy and associated with parturition [144]. This group reported melatonin values as high as 300 pg/mL fluid; these values are higher than nocturnal blood levels in young adult females or males. Subsequent studies found far lower concentrations of melatonin in amniotic fluid. Thus, Kivela and co-workers [145] claimed maximal values around 100 pg/mL in nighttime-collected amniotic fluid samples and lower levels when the fluid was obtained during the day, *i.e.*, there was a diurnal rhythm of melatonin concentrations in amniotic fluid reminiscent of that seen in the fetal and maternal blood, but at lower levels than in blood. Finally, the most recent report found melatonin values in the low pg/mL range in amniotic fluid collected after different durations of pregnancy [146].

While each of these groups noted the presence of melatonin in amniotic fluid, the measured amounts obviously varied widely. Whether the cells of the amniotic membrane produce melatonin seems not to have been investigated. In addition to its potential local production, melatonin in the amniotic fluid could be due to leakage from the placenta or fetal tissues. Functionally, melatonin at this site would likely have antioxidant and anti-inflammatory actions as it does elsewhere. These actions could be mediated by the typical melatonin membrane receptor, MT1, and also via direct free radical scavenging [90,147]. Attempts have been made to equate differences in amniotic fluid melatonin concentrations with fetal distress, toxin exposure, *etc*., but the findings are too few and inconsistent to warrant any firm conclusions [144,145,148].

### 3.4. Parturition

Human parturition, *i.e.*, the sustained spontaneous contractions of the uterus leading to cervical effacement and ultimately causing delivery of the offspring, occurs more frequently in the late night and early morning hours than at any other time of the day [149,150]. The physiological basis for this rhythm has not been satisfactorily explained although evolutionarily, it has been assumed that it may have been safer for both the mother and the offspring for this process to occur during darkness when predation was less. The relative incapacitation that occurs as a result of labor and delivery would have increased their vulnerability to predators during the daylight hours. In non-human primates, inverting the normal light:dark cycle causes peak parturition time to also shift, suggesting that the circadian rhythm of delivery is a light-sensitive event [151]. Not surprisingly, the late pregnancy nocturnal peak of uterine contractions at night in both humans and non-human primates prompted scientists to examine clock mechanisms that account for this cycle. If the rhythm of delivery is hormonally influenced, glucocorticoids and melatonin, both of which have a circadian component, would be expected to be involved [152,153].

Since the central circadian clock, the suprachiasmatic nucleus [154], and the pineal gland [155], the source of the endogenous melatonin rhythm, must be intact for peak parturition in nocturnally-active rats to take place at the normal time led to the assumption that the melatonin cycle may be somehow involved as a circadian gating signal for delivery of the offspring. This was further strengthened by the observation that injecting melatonin into pinealectomized rats at the appropriate circadian time re-initiated the normal peak birthing period [155].

Whether the information garnered from reports on the nocturnally-active rat apply to diurnal species such as the human still remain unknown. Initially, studies in diurnally active species focused on oxytocin as the potential mediator of circadian information in non-human primates [156]. Yet, the bulk of the published data noted there was no substantial increase in circulating oxytocin levels during human labor [157,158] and, moreover, parturition in the human can still occur in the absence of oxytocin from the neurohypophysis [159], although in these cases there may be locally-produced oxytocin in the uterus that aids contractions [160]. More recently, melatonin, due to the circadian nature of its secretion from the pineal gland, has been invoked as a potential molecule that determines nocturnal delivery of the offspring in humans at night.

With the aid of receptor autoradiography and a radioreceptor assay, Schlabritz-Loutsevitch *et al.*[161] documented the presence of high-affinity, G-coupled, melatonin membrane receptors on uterine myometrial cells collected from both pregnant and non-pregnant women. The same group subsequently found that the actions of melatonin and oxytocin on the uterine myometrium utilized the same intracellular signaling cascades, e.g., stimulation of phospholipase C, protein kinase C and myosin light chain kinase, to augment uterine smooth muscle contraction [162,163]. Thus, melatonin and oxytocin apparently act in synergy to induce contraction of the human uterine myometrium. As an example, in the presence of submaximal levels of oxytocin, adding supplemental melatonin (1 nmol/L) causes full contraction of uterine muscle cells *in vitro*. Melatonin was also reported to upregulate the gap junction protein, connexin 43, in uterine myocytes; this would enhance intercellular communication and ensure uniform contractions of the myometrium. Finally, both melatonin and oxytocin receptors were found to be simultaneously upregulated in the human uterus. The clear implication of these findings is that the nocturnal melatonin surge may participate in the temporal gating of the molecular events that contribute to the intensive and coordinated uterine contractions that ensures nocturnal delivery of human offspring in term pregnancies at night [164].

**Figure 9:**
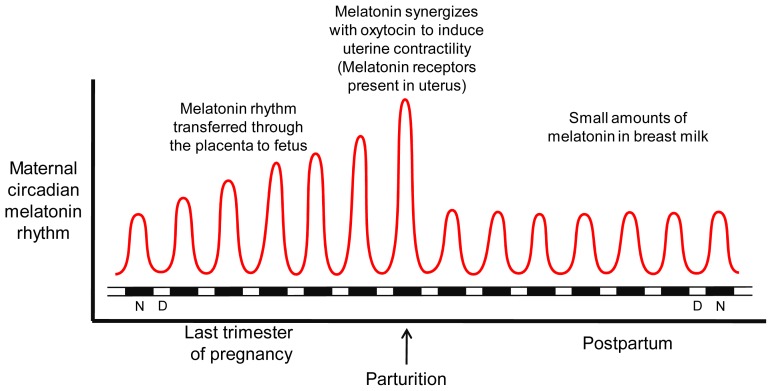
Diagrammatic representation of the circadian melatonin rhythm in human females during the last trimester of pregnancy and after delivery. The nocturnal melatonin peak gradually increases near term pregnancy. The augmented melatonin levels seem to aid in inducing uterine contractions at parturition since it has been shown to synergize with the released oxytocin to cause stronger uterine contractions. Shortly after delivery of the fetus, the nocturnal melatonin peak returns to its pre-pregnancy levels.

Olcese and co-workers [164] in a yet incomplete study have found that bright light exposure at night, which is known to suppress high nocturnal melatonin levels in humans [165], likewise impedes regular uterine contractions in late term human pregnancy. Collectively, the data accumulated to date are consistent with the melatonin rhythm being a pivotal factor in coordinating nocturnal myometrial contractions such that delivery of offspring more frequently occurs at night than during the day. Interestingly, human nocturnal melatonin levels increase in late pregnancy and reach a peak at the time of parturition [95,166] (Figure 9).

Olcese and colleagues [164] point out that in view of the genetic predisposition for the preterm delivery of the fetus by some women [167], it may be of value to examine the uterus of these females for melatonin receptor (also oxytocin receptor) polymorphisms in an attempt to provide an explanation for the early deliveries. There is additional information provided by these studies that may be useful. Namely, women with higher nocturnal melatonin surges may have more vigorous and coordinated uterine contractions at parturition. Also, perhaps women with a predilection for preterm delivery should avoid taking melatonin supplements during pregnancy. Due to the increasingly wide-spread use of artificial light at night with the concurrent suppression of melatonin, which is especially common in the hospital setting, the tendency for nocturnal delivery may continue to decrease. Finally, when oxytocin (e.g., Pitocin) is used to induce labor during the day, its efficacy may be enhanced if given in combination with melatonin thus making labor of shorter duration and delivery easier.

## 4. Melatonin: Male Reproductive Health

### 4.1. Sperm *in Situ*

Heavy metals and mercury in particular, are in general becoming increasingly common contaminants in the environment [168,169] and, when taken into cells, they damage them because of the induction of free radicals [170,171]. Rat spermatozoa, retrieved from the epididymis, dispersed in RPS medium and incubated with mercuric chloride exhibited a dose-dependent reduction in motility, a reduction in the activities of antioxidant enzymes, and an increased generation of H_2_O_2_ along with elevated levels of products of lipid peroxidation. When sperm samples were co-incubated with both mercuric chloride and melatonin (each at a concentration of 100 μM), all the changes induced by the heavy metal alone reverted back to the levels measured in the normal spermatozoa [172].

Other environmental contaminants also impair sperm function and fertility. Mice treated with the organophosphorus pesticide, diazinon, were found to have extensive DNA breakage and depressed chromatin packaging in the spermatozoa collected from the epididymis up to 32 days after the injection of the pesticide. When melatonin administration preceded the diazinon injection, the DNA damage imparted by the pesticide was markedly diminished [173]. Chemical pesticides, including organophosphates, are still extensively utilized in many countries and the fertility of the local fauna and of the humans who regularly use these compounds may be reduced [174,175]. Humans could possibly use supplements of melatonin to combat sperm (and other tissue) damage due to these agents.

Hypobaric hypoxia is experienced by individuals who ascend to a high altitude. Hypoxia is known to lead to the production of damaging free radicals. The exposure of mice to intermittent or continuous simulated high altitude (4500 meters above sea level) for 33 days resulted in increased teratozoospermia (teratospermia) as well as to elevated lipid peroxidation and DNA damage in the sperm [176]. The regular administration of melatonin under hypoxic conditions partially ameliorated the destructive actions of the low-oxygen environment. These findings have implications for not only ground-based individuals who move to high altitudes, but also to pilots who experience hypoxia when they fly at high altitudes [177].

### 4.2. Male Accessory Sex Organs

After being transported out of the testes, the spermatozoa are stored in the epididymis where they undergo further maturation. Peristaltic contractions of the smooth muscle and stereocilia on the luminal surface of the epithelial lining cells aid in the movement of the sperm through the epididymal ducts. The epithelial cells of the mammalian epididymis possess both the MT1 and MT2 membrane receptors and, judging from the effects of castration and hormone supplementation, their density is regulated by testosterone and hydrocortisone [178,179]. As in other organs, these receptors are coupled to a pertussis toxin sensitive G-proteins which are linked to the downregulation of cAMP. Via these membrane receptors, melatonin likely regulates the proliferation of the rat epididymal lining cells, although nuclear binding sites for melatonin in the epithelial cells have also been surmised [180].

Beyond its stimulation of epithelial cell proliferation in the epididymis, there is rather limited information on the benefits of melatonin in these organs. The harmful actions of homocysteine administration on epididymal sperm physiology along with the protective actions of melatonin have been examined [181]. After daily homocysteine injections into rats for 6 weeks, there was a measurable reduction in epididymal sperm concentration and in their motility. These actions were counteracted by melatonin treatment and, given that the indole elevated plasma antioxidative enzymes (not measured in the epididymis), the authors felt that the benefits of melatonin against homocysteine toxicity stemmed from its ability to reduce oxidative stress. Whether the antioxidative effects of melatonin in this study involved the receptors described above remains unknown.

Melatonin was also found to preserve total sperm concentrations and protect against morphologically abnormal sperm in the epididymis subjected to transient anoxia followed by re-oxygenation [182]. Ischemia was produced by clamping the testicular artery and vein for 1 h and then re-establishing the blood flow. Since ischemia/reperfusion damage is primarily a consequence of excessive free radical generation, melatonin’s ability to ameliorate damage to the epididymal spermatozoa was attributed to the direct free radical scavenging activity of the indoleamine.

Other accessory sex organs in males, with the exception of malignancies of the prostate, have been sparing investigated relative to the direct actions of melatonin on these tissues. For example, both the seminal vesicles and prostate exhibit marked changes in size and function in animals that are classified as photoperiod-sensitive seasonal breeders [183,184]. The changes in these organs are, however, secondary to alterations in androgen synthesis and secretion which, in turn, are regulated by pituitary gonadotropins. The changes at the hypothalamo-pituitary axis in these seasonal breeders is impelled by the seasonal fluctuations in the duration of the nightly melatonin message [63,65]. The annual changes in the nocturnal melatonin surge, however, seem not to directly impact either the seminal vesicles or prostate. As already mentioned, one exception to this general rule is experimental prostate cancers which are probably normally directly inhibited by melatonin [185,186]. This action of melatonin is considered outside the scope of the current review and the readership is directed to other sources of this information [187,188].

### 4.3. Protection of Ejaculated Animal Sperm

At least in the spermatozoa of the ram (*Rasa aragonesa*), the classic melatonin membrane receptors, MT1 and MT2, have been identified. By means of immunocytochemistry, four populations of receptors were tentatively defined [189]. Based on the distribution of the melatonin receptors, two subpopulations and two major populations of spermatozoa were detected. The minor groups showed receptor distribution over the entire head and tail while a second small subpopulation exhibited reactivity only in the sperm tail. The major subpopulation exhibited receptor reactivity over the equatorial, post-acrosomal, neck and tail regions while the last large group was reactive for the receptors at the equatorial region and tail. A reason for the heterogeneous distribution of the melatonin receptors among different groups was not deciphered. Western blot analyses were also consistent with the presence of both the MT1 and MT2 receptors in ram spermatozoa with evidence of heterodimerization of the receptors. This report provided no data regarding the downstream signaling mechanisms of the receptors.

There has been a rather long-standing interest in the potential use of melatonin to preserve sperm quality and viability during its storage. In one of the earliest studies, melatonin was subcutaneously implanted into donor rams and, thereafter, the quality of their ejaculated cryopreserved sperm was examined [190]. In frozen sperm of rams implanted during the breeding season, melatonin improved post-thaw viability and acrosome rates with no change in the mobility of the sperm. Damage to the sperm was also reduced since alkaline phosphatase release was significantly less from the sperm of melatonin-treated rams. When melatonin was implanted into rams during their non-breeding season, intact acrosome rates were enhanced and alkaline phosphatase release was reduced from post-thaw sperm. Clearly, donor sperm that are collected and cryopreserved seem to be functionally improved when the donor males have been treated with melatonin.

As a somewhat delayed follow-up to the study of Kaya *et al.*[190], Casao and colleagues [191] implanted rams (*Rasa aragonesa*) during the non-breeding season with melatonin and subsequently tested the motility of collected sperm using a computer-assisted sperm analysis system. Especially in rams that had implants for long duration (46–75 days), melatonin increased the percentage of progressive motile spermatozoa. In a second study, the ability of melatonin to influence the fertilizability of the sperm was examined with the aid of zona pellucida binding assay; this test revealed that sperm from melatonin-treated donors more readily attached to the oocyte. Finally, ewes inseminated with ejaculated sperm from donor rams exposed to melatonin exhibited an improvement in fertility and fecundity. The authors concluded that melatonin use can be an effective means to enhance sperm quality and improve their fertilizability.

One issue that their implantation studies did not resolve is whether the beneficial effects of melatonin on spermatozoa from melatonin-treated rams was due to direct actions on the sperm or a consequence of an alteration in the function of the hypothalamo-pituitary-gonadal axis. It had already been shown that exogenous melatonin from subcutaneous implants changes the pulsatile release frequency of hypothalamic gonadotropin-releasing hormone (GnRH) and elevates luteinizing hormone (LH), follicle stimulating hormone (FSH) and testosterone levels in rams [192–194]. Thus, the improvement in sperm quality in this study could have been due either to the direct actions of melatonin on the spermatozoa, e.g., as an antioxidant, or secondary to actions mediated by LH, FSH and especially testosterone. The presence of melatonin in ram seminal fluid is in higher concentrations than in serum of rams [195]; this is certainly consistent with the possibility that melatonin may have direct actions on the sperm.

Casao *et al.*[196] also investigated whether ejaculated ram sperm, incubated with melatonin, would impact sperm apoptotic-like changes and sperm quality as reflected in an *in vitro* fertilization (IVF) assessment. In this report, melatonin did not influence the kinematic parameters or viability of sperm; however, at a concentration of 1 μM melatonin decreased capacitation and phosphatidylserine translocation. Conversely, at 100 pM melatonin increased short-term capacitation which led to elevated oocyte fertilization rates in IVF. Moreover, the cleavage rate of oocytes fertilized by spermatozoa incubated in 100 pM was improved.

When stallion sperm was incubated at 37 °C for up to three hours in the absence or presence of melatonin (50, 100 or 200 pM or 1 μM), the sperm incubated with melatonin was noticeably improved compared to spermatozoa not exposed to melatonin [196]. This was reflected in a reduction in changes normally associated with apoptosis (increased sperm membrane permeability and depressed mitochondrial membrane potential), lower levels of products of lipid peroxidation and improved fluidity (less rigidity) of the spermatozoa plasma membranes. The peroxidation of lipids is considered to be a major hazard during the cryopreservation of sperm so its reduction by melatonin, along with the related functional improvements, is highly noteworthy. Using polyclonal antibodies and western blotting, Balao da Silva and co-workers [197] also documented the presence of both MT1 and MT2 receptors on stallion sperm. They predict that melatonin’s broad protective actions were a consequence of its direct free radical scavenging actions and its indirect functions in the stimulation of antioxidant enzymes (probably receptor-mediated).

The differential actions of melatonin on capacitation in this study were presumed to be related to different actions of melatonin. At 100 pM, melatonin presumably scavenged a sufficient number of radicals to protect the sperm and permit capacitation; at higher levels melatonin may have bound calmodulin and signaled capacitation, and the acrosome reaction [198,199]. The protective actions of melatonin against signs of apoptosis, e.g., phosphatidylserine translocation, were attributed to melatonin’s actions as a direct free radical scavenger and indirect antioxidant.

Spermatozoa of many species are cryopreserved and stored for prolonged periods. Freezing and thawing causes molecular damage and impairs the fertilizability of sperm. Considering this, Succu *et al.*[200] added melatonin (over a range of concentrations from 0.001 to 1 mM) to ram semen freezing extender fluid to determine if the presence of the indoleamine would ameliorate the damage and improve the quality of post-thawed sperm. At a 1 mM concentration, melatonin led to an improvement in all functional aspects of the sperm. Thus, melatonin in the sperm extender induced higher viability rates, a greater percentage of total motile and progressive motile spermatozoa, a larger number of sperm with greater average medium or rapid velocity, elevated intracellular ATP concentrations, and higher DNA integrity. Also, oocytes fertilized by melatonin-treated sperm exhibited higher total cleavage rates than did oocytes fertilized with ram sperm frozen in semen extender that lacked melatonin. Given that semen cryopreservation is associated with elevated levels of production of toxic reactants, including oxygen and nitrogen-based free radicals and associated molecules [201], it is likely that melatonin’s actions as an antioxidant contributed to its ability to mitigate against the damage to ram sperm and to improve their function.

Boar sperm is likewise often stored for an extended period prior to its use in artificial insemination. The usual storage temperature is 15–20 °C and the duration of storage is up to several days [202]. Melatonin, at a concentration of 1 μM was added to pig semen with the intent of enhancing the lifespan of refrigerated (at 17 °C) boar spermatozoa [203]. A computer-assisted sperm analysis system was used to evaluate a variety of sperm motility parameters in samples stored for 1, 4, or 7 days. Also, mitochondrial membrane potential, cell viability, membrane fluidity (which inversely correlates with the degree of lipid peroxidation [204]), and acrosome status were estimated using flow cytometry. By day 7, the number of static spermatozoa had increased and the percentage of progressive motile sperm was reduced. The velocity characteristics (curvilinear velocity, straight line velocity, and average path velocity) were elevated by melatonin treatment. The cytometric measures documented that melatonin increased the percentage of viable sperm with an intact acrosome and a significantly greater percentage of the sperm remained viable during the 7-day storage period. Melatonin did not change the mitochondrial membrane potential which remained the same as the sperm not treated with melatonin.

When the authors considered their collective data, Martin-Hidalgo and co-workers [203], concluded that melatonin had advanced boar spermatozoa to a hyperactive state. The hyperactivity in the early stages of storage may have been a result of an elevated synthesis of ATP; melatonin is known to promote mitochondrial complex efficiency and ATP production [205–207]. Due to its high lipid solubility, melatonin readily passes through the plasmalemma of cells and enters mitochondria [208]. Some of the actions of melatonin on sperm motility may have also been a consequence of its interaction with calmodulin [34,209] which, among several functions, influences cytoskeletal elements which may have impacted sperm motility. Despite the obvious positive effects, Martin-Hidalgo *et al.*[203] felt that melatonin did not significantly improve the function of stored boar sperm. The one exception was the percentage of live sperm with an intact acrosome. The study, albeit complete in terms of endpoints measured, only tested a single concentration of melatonin (1 μM) and whether the melatonin-treated sperm would have improved the rate of artificial insemination was not considered.

X- and Y-chromosome-bearing spermatozoa can be sorted by flow cytometry to produce the desired offspring in domestic animals [210]. The pregnancy rates from sperm segregated by this means, however, is lower than with normal sperm and the sexing process is believed to generate free radicals which compromise the function of the sperm [211]. Because of its high antioxidant activity, Li *et al.*[212] used melatonin as a potential protector of Nili-Ravi buffalo sperm during sex sorting. The authors included flow cytometry and laser tweezers Raman spectrometry to evaluate sperm quality after they had been incubated without or with melatonin (10^−4^ M) for up to 3 days at 27 °C. Melatonin supplementation improved mitochondrial activity of the sperm during sex sorting. Except for a single band at 1302/cm, the Raman spectra from sperm frozen after the addition of melatonin was significantly weaker. In summarizing their findings, Li and colleagues [212] argue that melatonin, due to its ability to effectively scavenge reactive oxygen species, is highly useful in protecting buffalo sperm during staining, sorting and freezing in semen extender. They also predicted that buffalo spermatozoa treated with melatonin would have an improved fertilization capability and increase pregnancy rates after embryo transfer or artificial insemination.

The hyperactive state of melatonin-incubated boar sperm [203] has also been observed in hamster spermatozoa [213]. For this study, the sperm were obtained from the caudal epididymis of adult Syrian hamsters; the samples were incubated in mTALP medium and, after isolation of the motile sperm, they were treated with either 1 pM or 1 μM melatonin. Hyperactive sperm, defined as those that exhibited asymmetric and whiplash flagellar movements or displayed a circular and/or octagonal swimming locus [214], were evaluated manually on images recorded with a CCD camera. Although the percentage of hamster sperm that become hyperactive after melatonin treatment significantly increased, the percentage of motile sperm did not. Normally, mammalian spermatozoa undergo capacitation, which includes the acrosome reaction and hyperactivation, in the female reproductive tract. Thus, Fujinoki [213] speculated that melatonin produced in the ovary served as the activating agent. Since melatonin is produced in several tissues of the female reproductive tract [95], however, the source of the melatonin that aids in hyperactivation of sperm in the uterus and/or fallopian tube remains undefined.

Mechanistically, Fujinoki [213] also found that treating hamster sperm with an MT1/MT2 melatonin receptor antagonist (luzindole), blocked the melatonin-mediated hyperactivation whereas incubation of sperm with two different MT2 antagonists failed to alter the hyperactive response. The presumption was that melatonin’s action on sperm hyperactivity was mediated by the MT1 receptor. Both the MT1 and MT2 melatonin receptors have been tentatively identified on the sperm midpiece [215–217]. The presence of the membrane melatonin receptors does not preclude the possibility that the indoleamine also functioned as a direct radical scavenger in spermatozoa.

### 4.4. Protection of Ejaculated Human Sperm

Since melatonin has been measured in human semen [218], Luboshitzky and co-workers [219] supposed that melatonin may impact human sperm quality. Accordingly, 8 men participate in a double-blind, cross-over study where the subjects were given 3 mg melatonin or placebo daily for 3 months. Sperm quality, as judged on the basis of concentration, motility and morphology, was unchanged in 6 men after melatonin treatment while in 2 men there was a reported decline in semen quality. Because of the many perceived shortcomings of this study, Lerchl [220] critically analyzed the data of Luboshitzky *et al.*[219] and provided strong arguments against the conclusion that melatonin compromised semen quality (in 2 of 8 men).

When the function of sperm mitochondria, like mitochondria in any cell, is compromised, they leak electrons from the respiratory chain leading to excessive free radical generation which damages these organelles and the sperm eventually undergo apoptosis. Antioxidants, and melatonin in particular [221,222], have often been found to ameliorate mitochondrial damage and cellular death under conditions of elevated oxidative stress. Moreover, oxidative damage generated in cells that is not mediated by faulty mitochondria is also overcome by melatonin. Shang *et al.*[222] tested this using human spermatozoa where free radicals were induced using a hypoxanthine/xanthine system. As indices of mitochondrial function, the authors measured the rise in mitochondrial succinate dehydrogenase activity and ROS generation and the reduction of mitochondrial membrane potential. The degree of change for each of these parameters was reduced in the presence of melatonin and the authors suggested the use of this antioxidant to protect sperm from oxidative damage.

When human sperm were incubated with 2 mM melatonin *in vitro* for 120 min and evaluated with a computer-assisted motility assessment device, the percentage of motile sperm, progressive motile sperm and rapid motile sperm were all elevated [223]. These changes were accompanied by a rise in sperm viability. Melatonin also reduced nitric oxide levels in the sperm (estimated from 4,5-diaminofluorescein-2/diacetate fluorescence) but no apparent change in ROS generation (estimated by 2,7-dichorofluorescein-diacetate fluorescence). Whether the benefits that melatonin provided for human spermatozoa were exclusively a result of its scavenging nitric oxide seems unlikely. Melatonin is known to stimulate antioxidant enzymes [93] that also could have aided in protecting the gametes. It is unlikely, however, that the concentrations of melatonin used in this study could be achieved *in vivo*.

In a series of studies from the same research groups [224–226], melatonin was tested for its ability to protect human sperm from undergoing apoptosis after their exposure to toxic agents. In their first study, human ejaculated sperm were treated with either a calcium mobilizing agonist or with an oxidizing agent, hydrogen peroxide. The endpoints included ROS generation, caspase-3 and caspase-9 activities, phosphatidylserine externalization and apoptosis. For sperm exposed to either the calcium mobilizing agonist or hydrogen peroxide, melatonin prevented all of the changes normally associated with apoptosis. The authors reminded the reader that the rationale for these studies stemmed from the observations that the conventional preparation techniques to preserve human sperm for assisted reproductive technologies promote ROS production and apoptosis induction. Thus, based on the findings uncovered, Espino *et al.*[224] suggested the possible use of melatonin, due to its antioxidant and antiapoptotic activities, as a component of the storage medium for sperm preservation.

**Figure 10:**
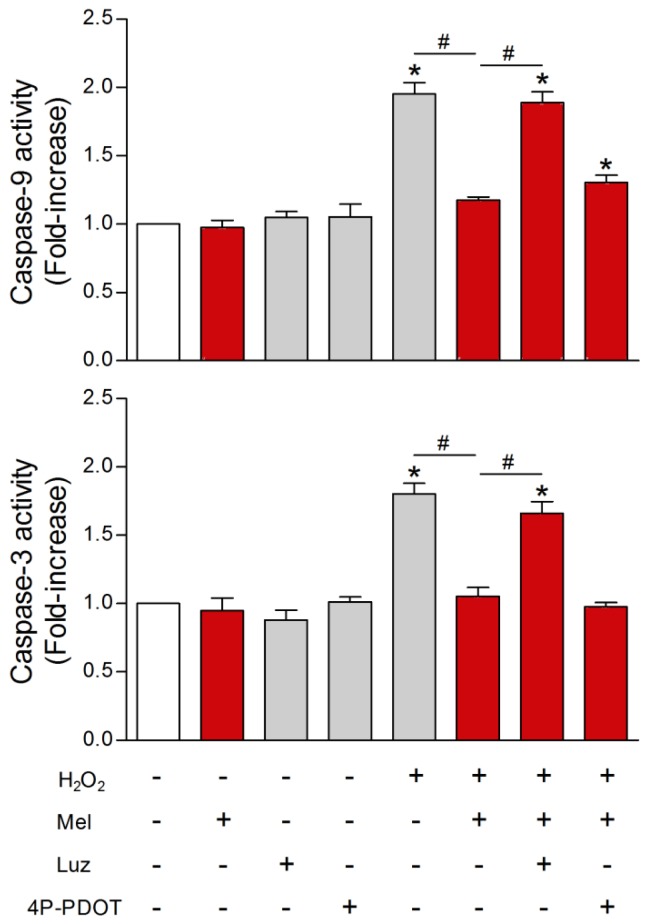
Evidence that the melatonin membrane receptor, MT1, is involved in the protective actions against H_2_O_2_ toxicity in human spermatozoa. Both the activity of caspase 9 (top panel) and caspase 3 (bottom panel), changes that are indicative of pending apoptosis, were elevated when the sperm were exposed to H_2_O_2_. These increases were blocked when luzindole (Luz), an MT1 and MT2 receptor antagonist, was added to the incubation medium but not when the selective MT2 blocker, 4P-PDOT, was added. These findings are consistent with the MT1 receptor mediating, at least in part, the ability of melatonin to defer H_2_O_2_-induced apoptotic processes in human spermatozoa. *p < 0.05, compared with all other values; # *p* < 0.05. From Espino *et al.*[225].

**Figure 11:**
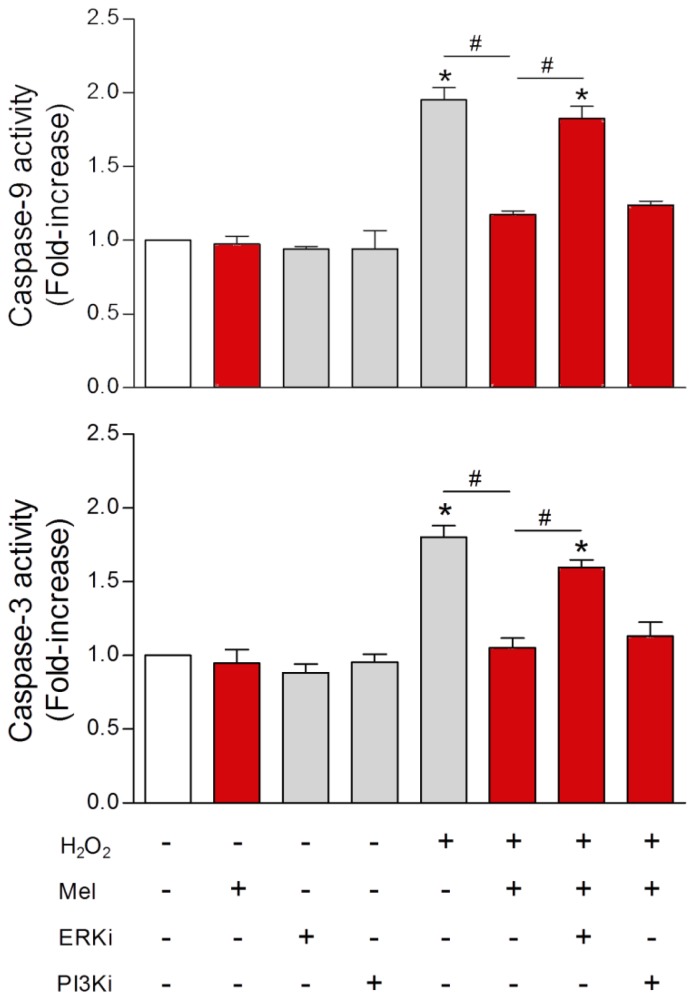
To define the signal transduction pathway relative to melatonin’s actions on human spermatozoa, sperm were exposed to H_2_O_2_ in the presence of either PD98059, an ERK inhibitor, or to LY294002, a selective pharmacological inhibitor of the P13K/Akt pathway. Clearly, suppressing ERK interfered with the H_2_O_2_-mediated rise in caspase 9 (**top panel**) and caspase 3 (**bottom panel**) activity indicating that this pathway is related to the ability of melatonin to forestall apoptotic processes in human spermatozoa. *p < 0.05 compared to all other values; # *p* < 0.05. From Espino *et al.*[225].

A subsequent report [225] was directed at identifying additional molecular events by which melatonin worked to improve human sperm integrity. With a combination of MT1 and MT2 melatonin membrane antagonists, it was found that only the former is involved in mediating melatonin’s effects (Figure 10). Additionally, the authors showed that melatonin stimulated the survival-promoting pathway extracellular signal-regulated kinase (ERK) in sperm thereby contributing to their survival; conversely, the PI3K pathway seemed not to be involved (Figure 11). With the use of the TUNEL assay, DNA fragmentation and apoptosis were found to be significantly inhibited as a consequence of melatonin administration.

**Figure 12:**
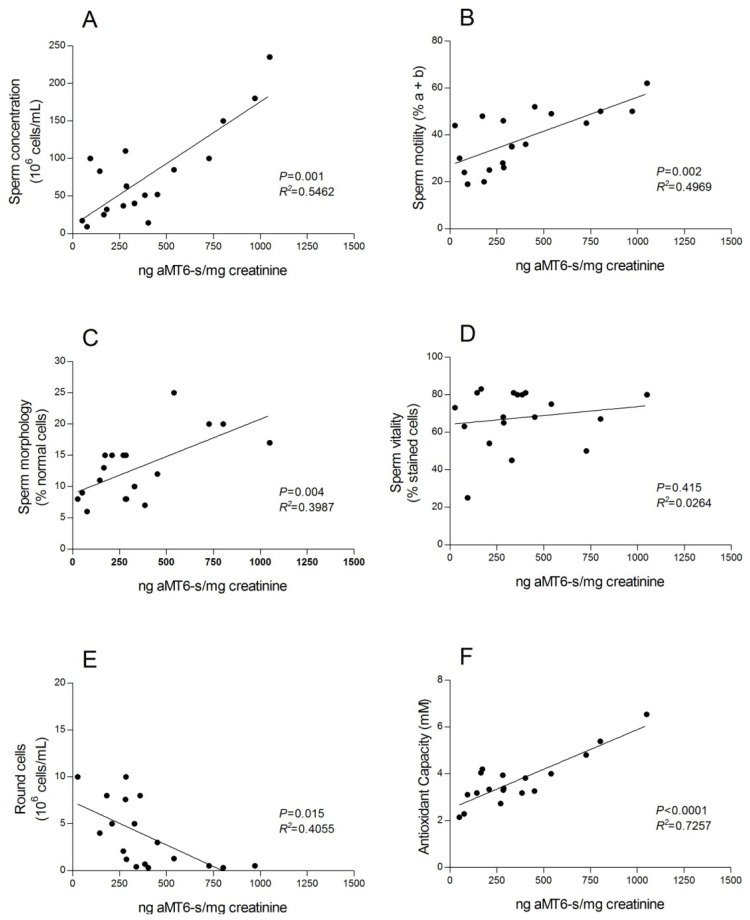
Correlations between urinary 6-hydroxymelatonin sulfate (aMT6-s), a major hepatic metabolite of melatonin, in the first urine morning void and various human sperm parameters in 20 adult males (20–40 years age). The semen samples were collected by masturbation after 4–5 days of sexual abstinence. The following parameters correlated positively with the concentration of urinary aMT6-s, which was taken as an index of endogenous melatonin levels: sperm concentration (**A**), sperm motility (**B**), sperm morphology (**C**), sperm vitality (**D**), and the total antioxidant status of the urine (estimated using the ABTS assay). There was a negative correlation between urinary aMT6-s and the number of round spermatids (cells) in the semen (**E**). Data from Espino *et al.*[225].

To examine the potential association between endogenous melatonin levels and sperm quality, ejaculated human semen from 52 men undergoing infertility counseling was evaluated and correlations were made with endogenous melatonin levels in each of the men. Melatonin estimations depended on the amounts of the chief melatonin metabolite, 6-sulfatoxymelatonin (aMT6-s) in the first morning urine void [226]. Urinary aMT6-s is commonly used as an index of endogenous melatonin levels in humans [227]. The concentrations of aMT6-s were found to positively correlate with sperm concentration, sperm motility, sperm morphology, and sperm vitality and negatively correlate with the number of round spermatids in the ejaculate (Figure 12). Uniformly, these findings are consistent with a positive effect of melatonin on sperm quality, at least in individuals who are potentially infertile. An obvious implication of the data garnered by Ortiz *et al.*[226] is that supplementing infertile men with melatonin may improve their fertility. Beyond this, the findings again emphasize the potential benefits of melatonin as an adjuvant in sperm preparation medium during assisted reproductive technologies.

## 5. Epilogue

Based on the reports summarized in this review, it seems obvious that melatonin is an essential component of optimal reproductive health. All levels of the reproductive axis are positively influenced by melatonin. Not all this melatonin is pineal-derived, since clearly several peripheral reproductive structures produce melatonin likely for their own use. The utilization of locally-produced melatonin by extra-pineal organs, and there are many that have this capability, may be a characteristic of all tissues. Indeed, we have recently speculated that mitochondria of all eukaryotic cells produce melatonin [228]. Obviously, if this conjecture is valid, there is no cell that does not synthesize this critically important indoleamine.

An important issue relates to the universe relationship between old age and melatonin levels. In both the pineal gland of animals [229,230] and the blood of all vertebrates including the human [231–233], melatonin concentrations wane as subject’s age. The decline in pineal melatonin synthesis is accompanied by a corresponding diminished melatonin production in peripheral organs as well [234]. The accompanying reduction in melatonin availability from both central and peripheral sites may contribute to the deterioration of reproductive physiology in the aged. An association between declining melatonin concentrations and a variety of age-related diseases/dysfunctions has already been confirmed in experimental animals [235–239]; to expect otherwise for the human would be short-sighted and perhaps irresponsible. On the basis of melatonin’s ability to reduce oxidative stress [23,37,90,240], damage that unequivocally compromises the function of the peripheral reproductive organs [80,241–243], a reduction in its protective effects due to the drop in endogenous melatonin production [232,234,244] with age would surely be expected to have a negative outcome. In humans, the total antioxidant capacity of the serum correlates with the melatonin concentrations in this fluid and both drop throughout aging [233].

The diminishment of melatonin’s antioxidative protection during aging could certainly compromise favorable cell physiology at multiple reproductive organ levels. Also, since melatonin passes the placenta [245,246] and has proven antioxidant actions in the fetus [247,248], it is possible that treatment of females with melatonin during pregnancy, especially when the pregnancy occurs late in the normal reproductive period, may reduce certain fetal problems associated with late-life pregnancies. Melatonin, even when given at extremely high doses to pregnant rats, has not been shown to have measureable untoward effects in either the mother of the fetus [249].

Finally, since the loss of melatonin may be part and parcel of reproductive decay during aging, an obvious corollary is that the daily administration of melatonin, e.g., to women approaching or entering menopause, may aid in prolonging reproductive health in terms of becoming pregnant and delivering healthy offspring [250].

The reports summarized in this survey emphasize the potential of melatonin in maintaining an optimally functioning peripheral reproductive system. Melatonin’s functions, both in terms of its receptor-mediated and receptor-independent (e.g., antioxidant) actions, are, however, ubiquitous. Thus, melatonin may be critical for not only conserving reproductive health, but health in general. Certainly, a variety of review articles have suggested the use of melatonin, alone or as an adjuvant therapy, to not only preserve good health but also possibly to defer or forestall some diseases normally associated advancing age [59,235–237,251–258]. In view of melatonin’s many benefits, it seems, as stated in the title of this review, that melatonin is ready for prime time, *i.e.*, to be used clinically. A number of human trials are on-going which the authors feel will certify the utility of this essential biogenic amine not only for reproductive well-being, but for the improved health of other tissues as well.

## Figures and Tables

**Table 1:** Melatonin, 3 mg daily, improved the clinical outcome of patients undergoing IVF-ET (*in vitro* fertilization + embryo transfer). One hundred and fifteen women (56 + 59) who had failed to become pregnant in a previous IVF-ET cycle were either supplemented with melatonin or not given the antioxidant.

	Melatonin Supplementation (56 patients)	No Melatonin Supplementation (59 patients)
Fertilization rate in a previous IVF-ET cycle	20.2% ± 19.0%	20.9% ± 16.5%
Fertilization rate	50.0% ± 38.0%	22.8% ± 19.0%
Pregnancy rate	11/56 (19.6%)	6/59 (10.2%)

Melatonin supplementation increased the rate of fertilization (20.2 *vs*. 50.0, *p* < 0.01). The pregnancy rate also doubled (these values were not statistically significantly different). Data from Tamura *et al.*[96].
